# Aiding the well celebrated Kuk's randomized response technique through auxiliary and prior information

**DOI:** 10.1016/j.heliyon.2024.e27546

**Published:** 2024-03-13

**Authors:** Zawar Hussain, Ishtiaq Hussain, Salman A. Cheema, Kalim Ullah, Sultan Salem, Walid Emam, Yusra Tashkandy

**Affiliations:** a Department of Statistics, Faculty of Computing, The Islamia University of Bahawalpur, Pakistan; b Department of Applied Mathematics, Chung Yuan Christian University, Taoyuan City, 32023, Taiwan; c Department of Applied Sciences, National Textile University, Faisalabad, Pakistan; d Foundation University Medical College, Foundation University School of Health Sciences, Pakistan; e Department of Economics, Birmingham Business School, College of Social Sciences, University of Birmingham, Edgbaston, Birmingham, England, B15 2TT, UK; f Department of Statistics and Operations Research, Faculty of Science, King Saud University, P.O. Box 2455, Riyadh, 11451, Saudi Arabia

**Keywords:** Auxiliary variable, Kuk design, Randomized response techniques, Sensitive attribute, Shrinkage estimation procedures

## Abstract

Asking direct questions in face to face surveys about sensitive traits is an intricate issue. One of the solutions to this issue is the randomized response technique (RRT). Being the most widely used indirect questioning technique to obtain truthful data on sensitive traits in survey sampling RRT has been applied in a variety of fields including behavioral science, socio-economic, psychological, epidemiology, biomedical, criminology, data masking, public health engineering, conservation studies, ecological studies and many others. This paper aims at exploring the methods to subsidize the randomized response technique through additional information relevant to the parameter of interest. Specifically, we plan to contribute by proposing more efficient hybrid estimators compared to existing estimator based on (Kuk, 1990) [31] family of randomized response models. The proposed estimators are based on the methodology of incorporating the pertinent information, available on the basis of either historical records or expert opinion. Specifically, in case of availability of auxiliary information, the regression-cum-ratio estimator is found to be the best to further enhance the estimation through (Kuk, 1990) [31] model while the (Thompson, 1968) [49] shrinkage estimation is observed to be yielding more precise and accurate estimator of sensitive proportion. The findings in this study signify the importance of the proposed methodology. Additionally, to support the mathematical findings, a detailed numerical investigation to evaluate the comparative performances is also conducted. Based on performance analysis, overwhelming evidences are witnessed in the favor of proposed strategies.

## 1 Introduction

In human population surveys, collecting data on sensitive or illicit behaviors through direct questioning generally results in falsified answers or refusal to respond. It is easy to understand that socially acceptable or desirable traits like helping energy conservation, pollution reduction, motivating elections participation and alike are expected to be over reported. On the other hand, socially unacceptable or undesirable traits like drunken driving, tax evasion, plagiarism, extra marital affairs, criminal frauds, using marijuana and many other similar behaviors are expected to be under reported. It is also possible, however, that some of the survey respondents refuse to report because of social stigma and fear of losing their privacy. Misreporting and non-response provoke biased and inefficient estimators and having a valid analysis becomes intricate. To cope with this problem of biased and inefficient estimation and questionable analysis a number of indirect data gathering tools have been developed to obtain truthful response from respondents by ensuring the protection against the privacy of the respondents. There are three major types of indirect questioning techniques, namely, the randomized response technique (RRT), the item count technique (ICT), and the non-randomized response technique (NRRT). The honor of publishing the first treatise on RRT fell to the [1] to estimate the proportion of a dichotomous variable, the ICT was initially coined by the [2] and NRRT was originally contributed by [50]. While the field of RRT has not kept pace in practice, it is interesting to mention that theory on indirect questioning techniques has grown phenomenally.

A more recent review of RRTs is given by Ref. [3] where they quite vividly mentioned that the RRT has had been one of the most commonly indirect questioning methods to procure information on sensitive traits. Motivated by the seminal work of [1], many have contributed in estimation of the proportion of sensitive attributes. The concept of RRT is based on providing a random mechanism to respondents in order to obtain a randomized response. Borrowing the concept of RRT many authors including [[4], [5], [6], [7], [8], [9], [10], [11], [12], [13], [14], [15], [16], [17], [18], [19], [20], [21], [22]] extended the work of Warner [1] and suggested improved techniques. The idea of RRT was further extended to estimate the mean of a sensitive variable by Refs. [23,[24], [25], [26],[11], [12], [27], [28]] and many others. A very brief overview of few among numerous noteworthy efforts is given in Refs. [29,30,9,31].

In face to face surveys, main advantage of the RRT over ICT and NRRT is that the reported response of the respondent cannot be traced back to the true response of the respondent. Moreover, in case of having more than one response from a given respondent, different responses may be reported. This is not the case when ICT and NRRT are applied. In addition, a respondent may be selected in the sample more than once in simple random sampling with replacement and every time his/her response would be different. Again, both the ICT and NRRT do not share this feature. That is why, we chose RRT for further enhancement.

[32] concluded in their study that RRT proposed by Ref. [13] is comparatively better than the [33] family of RRTs when efficiency and privacy protection of both the families of RRTs are compared. Amongst the many RRTs such as forced response The use of [13] RRT has also been advocated by Refs. [34,35,33] and more recently by Ref. [36]. Keeping in view the recommendations by Ref. [36], we decided to study [13] RRT to explore it further and making its use in survey sampling by enriching it with auxiliary and prior information.

In his preliminary work [13], argued that respondent may still feel insecure even when he/she is provided with randomization device; he urged to use two decks of cards each containing two types of cards with different colors, say red and green. A respondent is then directed to use, first (second), deck with proportion $\theta_{1}\left(\theta_{2}\right)$ of red cards. The *i*th respondent, either using the first or second deck, is asked to randomly draw a card and report the color of the card drawn without disclosing the deck he/she have used. Let $X_{i}\left(Y_{i}\right)$ be the color of the card drawn from the first (second) deck and let $Z_{i}$ be the reported response. Then $Z_{i}$ can be written as,

(1) $$Z_{i}=\alpha_{i}X_{i}+\left(1-\alpha_{i}\right)Y_{i}\text{,}$$

where, $Z_{i}∼Bernoulli\left(p\right),X_{i}∼Bernoulli\left(\theta_{1}\right),Y_{i}∼Bernoulli\left(\theta_{2}\right)\;and\;\alpha_{i}∼Bernoulli\left(\pi \right)\text{.}$ Thus, using equation (1), the expected response of the *i*th respondent is given as, $E\left(Z_{i}\right)=p=\pi \mu_{x}+\left(1-\pi \right)\mu_{y}\text{,}$ which is further simplified as,

(2) $$\bar{Z}=\pi \left(\mu_{x}-\mu_{y}\right)+\mu_{y}\text{,}$$

where, $\mu_{x}=\theta_{1}$ and $\mu_{y}=\theta_{2}$. By using equation (2), the unbiased moment estimator (high-dimensional) of proportion of sensitive attribute employing Kuk's design is then provided as,

(3) $${\hat{\pi }}_{kuk}=\frac{\bar{z}-\mu_{Y}}{\mu_{X}-\mu_{Y}}\text{.}$$

The variance of the estimator given in equation (3) is reported as,

(4) $$Var\left({\hat{\pi }}_{kuk}\right)=\frac{\pi \left(1-\pi \right)}{n}+\frac{\pi \sigma_{x}^{2}+\left(1-\pi \right)\sigma_{y}^{2}}{n{\left(\theta_{1}-\theta_{2}\right)}^{2}}\text{,}$$

where, $\sigma_{x}^{2}=\theta_{1}\left(1-\theta_{1}\right)$ and $\sigma_{y}^{2}=\theta_{2}\left(1-\theta_{2}\right)$

Besides obtaining the data on study variable of primary interest, it is sometimes possible to collect data on an auxiliary variable thought to be correlated with the study variable. The data on auxiliary variable is collected with no additional sampling cost and can be fruitfully used either at the sampling stage or the estimation stage. There exist numerous studies utilizing auxiliary information at the estimation stage. While applying RRT, many authors utilized auxiliary information to suggest further improved estimation of a sensitive mean or proportion. In this regards [37,38,39], are some of the authors to be mentioned amongst many. Similarly, some sort of prior information about the parameter of interest may also be available in some studies. This prior information may be a point guess or complete probability distribution [40,41,42,43]. studied the effect of relevant prior guess about the parameter of interest on the estimation and noted a positive contribution in the sense of reducing variability in the estimation. To our knowledge, there is no study available on improving the most celebrated Kuk's RRT by using auxiliary or prior information. To fill this gap and motivated by the use of auxiliary information and prior knowledge about the parameter of interest, we plan to further improve the most celebrated Kuk's design by using auxiliary as well as prior information. Building on the above mentioned preliminaries of [13] model, in this paper, we introduce methods to incorporate further relevant information in the study and thus aid the [13] model to estimate the proportions of sensitive attribute more precisely.

The objective of this paper is met by using auxiliary information as well as shrinkage estimation techniques to enhance the performance of Kuk's design. The role of auxiliary information in improving the coverage density and thus performance of estimator is well understood in survey research; see for example, [38,44]. The task of incorporating auxiliary information is fulfilled by employing, ratio estimator, product estimator, regression estimator and generalized regression-cum-ratio estimator. The performance of above mentioned estimators is then studied by calculating the relative efficiencies with respect to Kuk estimator given in equation (3). In using the relevant information other than obtained on auxiliary variable, many has argued highlighting the utility of shrinkage estimation techniques, either in the form of crude prior guess or based on expert opinion, to attain more practical estimates; see for example, [45,20]. The applicability of shrinkage estimators when the interest lies in the estimation of proportion of sensitive characteristics in a population is explored by employing two pioneer methods of the field; [46,47].

This paper is divided into six major sections. In section 2, we establish the mathematics to incorporate the auxiliary information in usual Kuk's design and thus aiding the study variable. The mathematical expressions for bias and MSE of estimators are derived. Section 3 presents a detailed performance analysis of auxiliary information based proposed estimators with respect to Ref. [13]. The shrinkage estimation procedures are employed in the estimation of proportions of sensitive attribute in section 4. Optimal ranges where shrinkage techniques remain efficient than [13] estimator are also derived and numerically evaluated for various combinations of pre-fix parametric values. Section 5, comprehends the numerical evaluations of performance analysis of shrinkage based methods with respect to Ref. [13] RRT. For this purpose, we considered optimal point and mid-point over the permissible ranges of weights (established in section 4) involving shrinkage estimation. Lastly, section 6 is devoted to general discussion and future research aspects.

## 2 Incorporating the auxiliary information

This section establishes the mathematics required to further evaluate the comparative performances of estimators and thus provides the grounds to explain the applicability of the proposed methods of including auxiliary information.

### 2.1 Ratio estimator

The ratio estimator can be written as,

(5) $${\bar{z}}_{R}=\bar{z}\left(\frac{\bar{W}}{\bar{w}}\right)\text{,}$$

where, $\bar{W}$ is the population mean of auxiliary variable (assumed to be known, see Ref. [48], page. 151)) and $\bar{w}$ is sample mean for auxiliary variable estimated through the sample under study. By using equation (5), the estimator of sensitive proportion using auxiliary information in the form of ratio estimator, ${\hat{\pi }}_{R}$, is then written as,

(6) $${\hat{\pi }}_{R}=\frac{{\bar{z}}_{R}-\mu_{Y}}{\mu_{X}-\mu_{Y}}\text{.}$$

#### 2.1.1 Bias of the estimator

Now, we derive the expressions to evaluate the bias of the estimator given in equation (6). For this purpose, equation (5) is written as,

(7) $${\bar{z}}_{R}=\bar{Z}\left(1+e_{0}\right){\left(1+e_{1}\right)}^{-1}\text{,}$$

where, $e_{0}=\frac{\bar{z}-\bar{Z}}{\bar{Z}}$ and $e_{1}=\frac{\bar{w}-\bar{W}}{\bar{W}}\text{.}$ By employing Taylor's series expansion and ignore higher order terms, equation (7) is further simplified as,

(8) $${\bar{z}}_{R}≃\bar{Z}+\bar{Z}\left(e_{0}-e_{1}+e_{1}^{2}-e_{0}e_{1}\right)\text{.}$$

By putting expression of equations (2), (8) into equation (6) and solving, we get,

(9) $${\hat{\pi }}_{R}-\pi ≃\frac{\bar{Z}\left(e_{0}-e_{1}+e_{1}^{2}-e_{0}e_{1}\right)}{\mu_{X}-\mu_{Y}}\text{.}$$

The bias in ratio estimator is then evaluated by applying expectation on both sides of equation (9), that is,

(10) $$E\left({\hat{\pi }}_{R}-\pi \right)=\bar{Z}\left(\frac{1-f}{n}\right)\frac{\left(C_{w}^{2}-\rho_{zw}C_{w}C_{z}\right)}{\mu_{X}-\mu_{Y}}\text{,}$$

where in the above equation (10), $C_{w}^{2}=\frac{\sigma_{w}^{2}}{{\bar{W}}^{2}}$, while $\sigma_{w}^{2}$ is population variance of auxiliary variable. In this study, the auxiliary variable is generated through U[0.1,0.5]. Similarly, $C_{Z}^{2}=\frac{\sigma_{z}^{2}}{{\bar{Z}}^{2}}$, where $\sigma_{z}^{2}=\pi \left(1-\pi \right){\left(\mu_{x}-\mu_{y}\right)}^{2}+\pi \sigma_{x}^{2}+\left(1-\pi \right)\sigma_{y}^{2}\text{,}$ is variance of randomized response and $\rho_{wz}$ denotes the correlation co-efficient for auxiliary variable and randomized response. Therefore,

(11) $$C_{z}^{2}=\frac{\pi \left(1-\pi \right){\left(\mu_{x}-\mu_{y}\right)}^{2}+\pi \sigma_{x}^{2}+\left(1-\pi \right)\sigma_{y}^{2}}{{\left(\pi \mu_{x}+\left(1-\pi \right)\mu_{y}\right)}^{2}}$$

By using equation (11) in equation (10), the final expression for the bias of the estimator is given in equation (12).

(12) $$Bias\left({\hat{\pi }}_{R}\right)=\bar{Z}\left(\frac{1-f}{n}\right)\frac{\left\{C_{w}^{2}-\rho C_{w}\frac{\sqrt{\pi \left(1-\pi \right){\left(\mu_{x}-\mu_{y}\right)}^{2}+\pi \sigma_{x}^{2}+\left(1-\pi \right)}\sigma_{y}^{2}}{\pi \mu_{x}+\left(1-\pi \right)\mu_{y}}\right\}}{\mu_{X}-\mu_{Y}}$$

It is important to note that the correlation between auxiliary variable and randomized response, technically, cannot be controlled by the researcher. Therefore, to match the practical limitations of the design, we incorporated this co-efficient by exploiting the relationship between auxiliary variable and study variable; it is achieved by rewriting the correlation co-efficient, $\rho_{zw}$, in terms of $\rho_{\alpha w}$. Mathematically,

$$\rho_{zw}=\frac{\text{cov}\left(Z,W\right)}{\sqrt{\text{var}\left(Z\right)}\sqrt{\text{var}\left(W\right)}}=\frac{\mu_{X}\text{cov}\left(\alpha ,W\right)+\mu_{Y}\text{cov}\left(\left(1-\alpha \right),W\right)}{\sqrt{\left(\pi \sigma_{x}^{2}+\left(1-\alpha \right)\sigma_{y}^{2}\right)\sigma_{W}^{2}}}$$

$$\rho_{zw}=\frac{\mu_{X}\text{cov}\left(\alpha ,W\right)}{\sigma_{\alpha }\sigma_{W}\sqrt{\left(\frac{\sigma_{x}^{2}}{1-\pi }+\frac{\sigma_{y}^{2}}{\pi }\right)}}+\frac{\mu_{Y}\text{cov}\left(\left(1-\alpha \right),W\right)}{\sigma_{\alpha }\sigma_{W}\sqrt{\left(\frac{\sigma_{x}^{2}}{1-\pi }+\frac{\sigma_{y}^{2}}{\pi }\right)}}=\frac{\rho_{\alpha w}\left(\mu_{X}-\mu_{Y}\right)}{\sqrt{\left(\frac{\sigma_{x}^{2}}{1-\pi }+\frac{\sigma_{y}^{2}}{\pi }\right)}}$$

which is then simplified as,

(13) $$\rho_{zw}=\frac{\rho_{\alpha w}\sigma_{\alpha }\left(\mu_{X}-\mu_{Y}\right)}{\sigma_{Z}}\text{,}$$

where, $\sigma_{\alpha }^{2}=\pi \left(1-\pi \right)$

#### 2.1.2 Mean square error of the estimator

The general expression to calculate MSE of the estimator is,

(14) $$MSE\left({\hat{\pi }}_{R}\right)=\frac{MSE\left({\bar{z}}_{R}\right)}{{\left(\mu_{X}-\mu_{Y}\right)}^{2}}\text{,}$$

where, $MSE\left({\bar{z}}_{R}\right)=E{\left({\bar{z}}_{R}-\bar{Z}\right)}^{2}$. It is easily verifiable that, by substitution and expansion, we get, $MSE\left({\bar{z}}_{R}\right)≃E{\left({\bar{z}}_{R}-\bar{Z}\right)}^{2}≃{\bar{Z}}^{2}E\left(e_{0}^{2}+e_{1}^{2}-2e_{0}e_{1}\right)={\bar{Z}}^{2}\left(\frac{1-f}{n}\right)\left(C_{w}^{2}+C_{z}^{2}-2\rho_{wz}C_{w}C_{z}\right)$.

By using this in equation (14), we obtain,

(15) $$MSE\left({\hat{\pi }}_{R}\right)={\bar{Z}}^{2}\left(\frac{1-f}{n}\right)\frac{\left(C_{w}^{2}+C_{z}^{2}+2\rho_{wz}C_{w}C_{z}\right)}{{\left(\mu_{X}-\mu_{Y}\right)}^{2}}\text{.}$$

Using equation (11) in (15), we obtain the explicit expression for MSE of the ratio estimator for sensitive proportion as

$$MSE\left({\hat{\pi }}_{R}\right)=\frac{{\bar{Z}}^{2}\left(\frac{1-f}{n}\right)}{{\left(\mu_{X}-\mu_{Y}\right)}^{2}}\{\frac{\pi \left(1-\pi \right){\left(\mu_{x}-\mu_{y}\right)}^{2}+\pi \sigma_{x}^{2}+\left(1-\pi \right)\sigma_{y}^{2}}{{\left(\pi \mu_{x}+\left(1-\pi \right)\mu_{y}\right)}^{2}}$$

$$+C_{w}^{2}-2\rho_{wz}C_{w}\frac{\left\{C_{w}^{2}-\rho C_{w}\frac{\sqrt{\pi \left(1-\pi \right){\left(\mu_{x}-\mu_{y}\right)}^{2}+\pi \sigma_{x}^{2}+\left(1-\pi \right)}\sigma_{y}^{2}}{\pi \mu_{x}+\left(1-\pi \right)\mu_{y}}\right\}}{\mu_{X}-\mu_{Y}}\}$$

### 2.2 Product estimator

Another most commonly used estimator incorporating additional information to support the study under consideration was introduced by Murthy [49] and is known as product estimator in the literature. The estimator is written as,

$${\bar{z}}_{p}=\bar{z}\left(\frac{\bar{w}}{\bar{W}}\right)\text{,}$$

where, $\bar{W}$, the population mean of auxiliary variable is considered to be known. Employing this form of estimator, the estimator of proportion of sensitive characteristic is written as,

$${\hat{\pi }}_{P}=\frac{{\bar{z}}_{P}-\mu_{Y}}{\mu_{X}-\mu_{Y}}\text{.}$$

#### 2.2.1 Bias of the estimator

The bias of product estimator is found by following the same steps as for ratio estimator in above sub-section. The expression of bias is,

$$Bias\left({\hat{\pi }}_{P}\right)=\bar{Z}\left(\frac{1-f}{n}\right)\frac{\left\{C_{w}^{2}+\rho C_{w}\frac{\sqrt{\pi \left(1-\pi \right){\left(\mu_{x}-\mu_{y}\right)}^{2}+\pi \sigma_{x}^{2}+\left(1-\pi \right)}\sigma_{y}^{2}}{\pi \mu_{x}+\left(1-\pi \right)\mu_{y}}\right\}}{\mu_{X}-\mu_{Y}}$$

#### 2.2.2 Mean square error of the estimator

The MSE of product estimator is found on same lines as for ratio estimator; it is trivial to verify that the MSE of product estimator can be quantified by using equation given below;

$$MSE\left({\hat{\pi }}_{P}\right)=\frac{{\bar{Z}}^{2}\left(\frac{1-f}{n}\right)}{{\left(\mu_{X}-\mu_{Y}\right)}^{2}}\{\frac{\pi \left(1-\pi \right){\left(\mu_{x}-\mu_{y}\right)}^{2}+\pi \sigma_{x}^{2}+\left(1-\pi \right)\sigma_{y}^{2}}{{\left(\pi \mu_{x}+\left(1-\pi \right)\mu_{y}\right)}^{2}}+C_{w}^{2}+2\rho_{wz}C_{w}\frac{\left\{C_{w}^{2}-\rho C_{w}\frac{\sqrt{\pi \left(1-\pi \right){\left(\mu_{x}-\mu_{y}\right)}^{2}+\pi \sigma_{x}^{2}+\left(1-\pi \right)}\sigma_{y}^{2}}{\pi \mu_{x}+\left(1-\pi \right)\mu_{y}}\right\}}{\mu_{X}-\mu_{Y}}\}$$

### 2.3 Regression (difference) estimator

Regression (difference) estimator is another estimator capable of incorporating auxiliary information and thus enhances the utility of estimator by using additional information. The general from of regression (difference) estimator is given as,

$${\bar{z}}_{\text{Re}g}=\bar{z}+b\left(\bar{W}-\bar{w}\right)\text{,}$$

where, b is a constant projecting the extent of influence of auxiliary information induced in the study. By using this estimator, the estimator of proportion of sensitive characteristics takes the form as,

(16) $${\hat{\pi }}_{\text{Re}g}=\frac{{\bar{z}}_{\text{Re}g}-\mu_{Y}}{\mu_{X}-\mu_{Y}}\text{.}$$

#### 2.3.1 Bias of the estimator

It can be verified that by expanding and further simplifying, ${\bar{z}}_{\text{Re}g}$, results as under,

(17) $${\bar{z}}_{\text{Re}g}=\bar{Z}+\bar{Z}\left(e_{0}\right)-b\left(e_{1}\right)\bar{W}\text{.}$$

By putting equation (17) into equation (16), we attain,

$${\hat{\pi }}_{\text{Re}g}=\frac{\bar{Z}+\bar{Z}\left(e_{0}\right)-b\left(e_{1}\right)\bar{W}-\mu_{Y}}{\mu_{X}-\mu_{Y}}\text{.}$$

On substituting the value of $\bar{Z}$ provided by equation (2) in above equation, we get,

$${\hat{\pi }}_{\text{Re}g}=\frac{\pi \left(\mu_{X}-\mu_{Y}\right)+\mu_{Y}+\bar{Z}\left(e_{0}\right)-b\left(e_{1}\right)\bar{W}-\mu_{Y}}{\mu_{X}-\mu_{Y}}\text{.}$$

By taking expectation on both side of the above equation, we quantify the bias of the ${\hat{\pi }}_{\text{Re}g}$. It can be confirmed that,

(18) $$E\left({\hat{\pi }}_{\text{Re}g}-\pi \right)=0\text{.}$$

The above equation establishes the fact that regression estimation leads towards an unbiased estimator of ${\hat{\pi }}_{\text{Re}g}$.

#### 2.3.2 Mean square error of the estimator

The expression for evaluation of MSE of the sensitive proportion estimator applying regression (difference) estimator is written as,

(19) $$MSE\left({\hat{\pi }}_{\text{Re}g}\right)=\frac{Var\left({\bar{z}}_{\text{Re}g}\right)}{{\left(\mu_{X}-\mu_{Y}\right)}^{2}}\text{.}$$

$$Var\left({\bar{z}}_{\text{Re}g}\right)=E{\left(\bar{Z}\left(e_{0}\right)-b\left(e_{1}\right)\bar{W}\right)}^{2}\text{,}$$

which, then, can be simplified as,

(20) $$Var\left({\bar{z}}_{\text{Re}g}\right)=\left(\frac{1-f}{n}\right)\left({\bar{Z}}^{2}C_{Z}^{2}+b^{2}{\bar{W}}^{2}C_{W}^{2}-2b\rho_{zw}C_{W}C_{Z}\bar{Z}\bar{W}\right)\text{,}$$

where, $\rho_{zw}$ can be quantified by exploiting the relation given in equation (13). The optimal value of b is obtained by differentiating equation (20) with respect to b and equating it equal to zero. The optimal value is found through expression below,

(21) $$b_{opt}=\frac{\rho_{zw}\bar{Z}C_{z}}{\bar{W}C_{w}}\text{.}$$

Using this optimal value in equation (16), we get,

$$Var\left({\bar{z}}_{\text{Re}g}\right)=\left(\frac{1-f}{n}\right)\left({\bar{Z}}^{2}C_{z}^{2}+{\left(\frac{\rho_{zw}\bar{Z}C_{z}}{\bar{W}C_{w}}\right)}^{2}C_{w}^{2}-2\left(\frac{\rho_{zw}\bar{Z}C_{z}}{\bar{W}C_{w}}\right)\rho_{zw}\bar{Z}\bar{W}C_{z}C_{w}\right)\text{,}$$

which, further simplifies to,

$$Var\left({\bar{z}}_{\text{Re}g}\right)=\left(\frac{1-f}{n}\right){\bar{Z}}^{2}C_{z}^{2}\left(1-{\rho_{zw}}^{2}\right)\text{.}$$

By putting above resulting expression in equation (19), the MSE of ${\hat{\pi }}_{\text{Re}g}$ is derived as,

(22) $$Var\left({\hat{\pi }}_{\text{Re}g}\right)=\left(\frac{1-f}{n}\right)\frac{\left(\pi \left(1-\pi \right){\left(\mu_{x}-\mu_{y}\right)}^{2}+\pi \sigma_{x}^{2}+\left(1-\pi \right)\sigma_{y}^{2}\right)\left(1-{\rho_{zw}}^{2}\right)}{{\left(\mu_{X}-\mu_{Y}\right)}^{2}}\text{.}$$

It is important to note that when the optimum value of *d* is evaluated by equation (21), the suggested estimator is typically called the difference estimator. If all or some of the parameters on right hand side of equation (21) are unknown, the optimum *d* is unknown and estimator in equation (16) makes no sense. These unknown parameters are estimated from samples and thereby, optimum value of *d* is estimated from the sample. If estimated optimum value of *d* is used in equation (16), it is a regression (difference) estimator and its bias and MSE are exactly equal to expressions given in equations (18), (22), respectively.

### 2.4 Generalized regression-cum-ratio estimator

More recently, yet another estimator, generalized regression-cum-ratio estimator, capable of using additional information in the study was introduced by Ref. [50]. Without loss of generality, we write the estimator as,

(23) $${\bar{z}}_{Grr}=\left(k_{1}\bar{z}+k_{2}\left(\bar{W}-\bar{w}\right)\right)\left(\frac{\bar{W}}{\bar{w}}\right)\text{.}$$

Based on estimator given in equation (23), estimator of proportion of sensitive characteristic is rewritten as,

(24) $${\hat{\pi }}_{Grr}=\frac{{\bar{z}}_{Grr}-\mu_{Y}}{\mu_{X}-\mu_{Y}}\text{.}$$

#### 2.4.1 Bias of the estimator

The bias of the estimator is found by writing ${\bar{z}}_{Grr}$ as,

$${\bar{z}}_{Grr}=\left(k_{1}\bar{Z}+k_{1}\bar{Z}e_{o}-k_{2}\bar{W}e_{1}\right){\left(1+e_{1}\right)}^{-1}\text{,}$$

which, then, on expansion yields the expression as,

$${\bar{z}}_{Grr}-\bar{Z}=\bar{Z}\left(k_{1}-1\right)-k_{1}\bar{Z}e_{1}+k_{1}\bar{Z}e_{1}^{2}+k_{1}\bar{Z}e_{o}-k_{1}\bar{Z}e_{o}e_{1}-k_{2}\bar{W}e_{1}+k_{2}\bar{W}e_{1}^{2}\text{.}$$

By taking expectation on both sides of the above equation and putting into equation (24), bias of the estimator is written as,

(25) $$Bias\left({\hat{\pi }}_{Grr}\right)=\frac{\bar{Z}\left(k_{1}-1\right)+\left(\frac{1-f}{n}\right)\left(k_{1}\bar{Z}C_{w}^{2}-k_{1}\bar{Z}\rho_{zw}C_{w}C_{z}+k_{2}\bar{W}C_{w}^{2}\right)}{\mu_{X}-\mu_{Y}}\text{.}$$

#### 2.4.2 Mean square error of the estimator

The general expression of MSE is written as,

(26) $$MSE\left({\hat{\pi }}_{Grr}\right)=\frac{MSE\left({\bar{z}}_{Grr}\right)}{{\left(\mu_{X}-\mu_{Y}\right)}^{2}}\text{.}$$

On further solving $MSE\left({\bar{z}}_{Grr}\right)$, we obtained,

$$MSE\left({\bar{z}}_{Grr}\right)=E\{{\bar{Z}}^{2}{\left(k_{1}-1\right)}^{2}+k_{1}^{2}{\bar{Z}}^{2}e_{1}^{2}+k_{1}^{2}{\bar{Z}}^{2}e_{o}^{2}+k_{2}^{2}{\bar{W}}^{2}e_{1}^{2}+2{\bar{Z}}^{2}k_{1}^{2}e_{1}^{2}-2{\bar{Z}}^{2}k_{1}e_{1}^{2}-2{\bar{Z}}^{2}k_{1}^{2}e_{o}e_{1}$$

$$+2{\bar{Z}}^{2}k_{1}e_{o}e_{1}+2\bar{Z}\bar{W}k_{1}k_{2}e_{1}^{2}-2\bar{Z}\bar{W}k_{2}e_{1}^{2}-2k_{1}^{2}{\bar{Z}}^{2}e_{o}e_{1}+2k_{1}k_{2}\bar{Z}\bar{W}e_{1}^{2}-2k_{1}k_{2}\bar{Z}\bar{W}e_{o}e_{1}\}\text{,}$$

where, $\lambda =\frac{1-f}{n}\text{.}$ It further simplifies to,

$$MSE\left({\bar{z}}_{Grr}\right)=\{{\bar{Z}}^{2}{\left(k_{1}-1\right)}^{2}+k_{1}^{2}{\bar{Z}}^{2}\lambda \left(C_{z}^{2}+3C_{w}^{2}-4\rho_{zw}C_{z}C_{w}\right)+k_{2}^{2}{\bar{W}}^{2}\lambda C_{w}^{2}$$

(27) $$-2{\bar{Z}}^{2}k_{1}\lambda \left(C_{z}^{2}-\rho_{zw}C_{z}C_{w}\right)-2\bar{Z}\bar{W}k_{1}k_{2}\lambda \left(\rho_{zw}C_{z}C_{w}-2C_{w}^{2}\right)-2\bar{Z}\bar{W}k_{1}k_{2}\lambda C_{w}^{2}\}\text{.}$$

By differentiating equation (27) and equating the resulting quantity to zero, we attained optimal values of $k_{1}$ and $k_{2}$, such as,

(28) $$k_{1\left(opt\right)}=\frac{1-\lambda C_{w}^{2}}{1-\lambda \left(C_{w}^{2}-C_{z}^{2}\left(1-{\rho_{zw}}^{2}\right)\right)}\;\text{and}\;k_{2\left(opt\right)}=\frac{\bar{Z}}{\bar{W}}\left(1+k_{1\left(opt\right)}\left(\frac{\rho_{zw}C_{z}}{C_{w}}-2\right)\right)\text{.}$$

By using the values of $k_{1\left(opt\right)}$ and $k_{2\left(opt\right)}$, in equation (28), the expression of MSE is found as follows,

(29) $$MSE\left({\hat{\pi }}_{Grr}\right)≅\frac{{\bar{Z}}^{2}C_{z}^{2}\left(1-\rho_{zw}^{2}\right)\lambda \left(1-\lambda C_{w}^{2}\right)}{{\left(\mu_{X}-\mu_{Y}\right)}^{2}\left(C_{z}^{2}\left(1-\rho_{zw}^{2}\right)\lambda +\left(1-\lambda C_{w}^{2}\right)\right)}\text{.}$$

The use of equation (29) in equation (24), enables us to attain the final expression of $MSE\left({\hat{\pi }}_{Grr}\right)\text{,}$ as under,

$$MSE\left({\hat{\pi }}_{Grr}\right)≅\frac{A}{B}\text{,}$$

where, $A={\bar{Z}}^{2}\left(\frac{\pi \left(1-\pi \right){\left(\mu_{x}-\mu_{y}\right)}^{2}+\pi \sigma_{x}^{2}+\left(1-\pi \right)\sigma_{y}^{2}}{{\left(\pi \mu_{x}+\left(1-\pi \right)\mu_{y}\right)}^{2}}\right)\left(1-\rho_{zw}^{2}\right)\lambda \left(1-\lambda C_{w}^{2}\right)$ and

$$B={\left(\mu_{X}-\mu_{Y}\right)}^{2}\left(\left(\frac{\pi \left(1-\pi \right){\left(\mu_{x}-\mu_{y}\right)}^{2}+\pi \sigma_{x}^{2}+\left(1-\pi \right)\sigma_{y}^{2}}{{\left(\pi \mu_{x}+\left(1-\pi \right)\mu_{y}\right)}^{2}}\right)\left(1-\rho_{zw}^{2}\right)\lambda +\left(1-\lambda C_{w}^{2}\right)\right)$$

## 3 Mathematical and numerical evaluation of the performance of estimators whileIncorporating auxiliary information

This section is dedicated to derive the mathematical expressions demonstrating the performance of considered estimators with respect to the estimator based on [13] model. Furthermore, we numerically evaluate the performance of the proposed estimators with respect to Ref. [13] estimator for various combinations of parameters involved. A detailed performance analysis is conducted to demonstrate the applicability of suggested estimators while fixing the population proportion of sensitive attribute π at different levels (0.1, 0.2, 0.3 and 0.4). For each level of π then, $\theta_{1}$ (defined in section 1) is allowed to take values 0.1, 0.2 and 0.3 and under each value of $\theta_{1}$, $\theta_{2}$ (defined in section 1) is employed taking the values equal to 0.6, 0.7, 0.8 and 0.9. Moreover, using all above combinations of parameters, performance of ratio and product estimators, with respect to Ref. [13], are explored over respective optimal range of correlation coefficient, $\rho_{zw}$, that is, $0.6\leq \rho_{zw}\leq 0.9$ for ratio estimator and $-0.9\leq \rho_{zw}\leq -0.6$ for product estimator; see for example [48], pages. 151 and 186. Additionally, the efficiency investigation for regression and generalized regression-cum-ratio estimators is conducted considering the $\rho_{zw}$ values equal to [±0.2,±0.4,±0.6,±0.7,±0.8±0.9].

### 3.1 *Comparing*${\hat{\pi }}_{kuk}$*and*${\hat{\pi }}_{R}$

The proposed estimator ${\hat{\pi }}_{R}$ is considered more efficient than usual [13] ${\hat{\pi }}_{kuk}$ estimator if,

(30) $$MSE\left({\hat{\pi }}_{kuk}\right)-MSE\left({\hat{\pi }}_{R}\right)\geq 0\text{,}$$

By employing equation (4) and equation (13), we get

$$\frac{{\bar{Z}}^{2}\left(\frac{1-f}{n}\right)C_{z}^{2}}{{\left(\mu_{x}-\mu_{y}\right)}^{2}}-\frac{{\bar{Z}}^{2}\left(\frac{1-f}{n}\right)\left(C_{w}^{2}+C_{z}^{2}-2\rho_{wz}C_{w}C_{z}\right)}{{\left(\mu_{x}-\mu_{y}\right)}^{2}}\geq 0\text{.}$$

On further simplification, we get the efficiency condition as,

$$\rho_{zw}\geq \frac{1}{2}\left(\frac{C_{w}}{C_{z}}\right)\text{.}$$

Thus, in the cases where inequality (34) holds, ${\hat{\pi }}_{R}$ is relatively more efficient than ${\hat{\pi }}_{kuk}$. Interestingly, if $C_{w}\approx C_{z}$, the above inequality reduces to $\rho_{zw}\geq \frac{1}{2}\text{,}$ which then demonstrate the fact that if auxiliary variable and randomized response are strongly and positively correlated the proposed ratio estimator always performs better than the existing estimator of [13]. This is in fact in agreement with the established theory of ratio estimator. Table 1, summarizes the numerical findings for various combinations of parameters over the permissible range of correlation coefficient. It can be noticed, in case of increasing value of correlation coefficient (strong positive correlation), the ratio estimator dominates the usual [13] estimator. Furthermore, yet another interesting finding aligned with theory is witnessed; as the value of randomization device parameter, π, increases the ratio estimator although remains efficient over optimal range but with downward trajectory. It is also observed that the difference between $\theta^{\prime s}$ has minimal effect on the comparative performance of both estimators.

**Table 1:** Performance analysis of ${\hat{\pi }}_{R}$ with respect to ${\hat{\pi }}_{kuk}$, in terms of *RE*.

ππ			$\begin{array}{l} \rho_{zw}\\ \theta_{1}\\ \theta_{2} \end{array}$$\rho_{zw}$			
	$\theta_{1}$	$\theta_{2}$	0.6	0.7	0.8	0.9
0.1	0.1	0.6	1.5024	1.7170	2.0031	2.4037
		0.7	1.5571	1.8454	2.2648	2.9308
		0.8	1.5768	1.9589	2.5857	3.8022
		0.9	1.4755	1.9337	2.8049	5.1044
	0.2	0.6	1.5091	1.7309	2.0291	2.4513
		0.7	1.5618	1.8597	2.2980	3.0065
		0.8	1.5741	1.9672	2.6221	3.9305
		0.9	1.4482	1.9075	2.7936	5.2167
	0.3	0.6	1.5158	1.7450	2.0558	2.5013
		0.7	1.5661	1.8738	2.3319	3.0866
		0.8	1.5700	1.9739	2.6576	4.0660
		0.9	1.4160	1.8734	2.7673	5.2926
0.2	0.1	0.6	1.4674	1.6495	1.8833	2.1943
		0.7	1.5223	1.7591	2.0832	2.5537
		0.8	1.5661	1.8738	2.3319	3.0866
		0.9	1.5741	1.9672	2.6221	3.9305
	0.2	0.6	1.4815	1.6760	1.9293	2.2727
		0.7	1.5349	1.7878	2.1405	2.6665
		0.8	1.5731	1.9012	2.4021	3.2616
		0.9	1.5640	1.9784	2.6915	4.2082
	0.3	0.6	1.4955	1.7032	1.9778	2.3581
		0.7	1.5466	1.8166	2.2010	2.7915
		0.8	1.5774	1.9267	2.4748	3.4587
		0.9	1.5460	1.9796	2.7514	4.5095
0.3	0.1	0.6	1.4314	1.5860	1.7779	2.0227
		0.7	1.4815	1.6760	1.9293	2.2727
		0.8	1.5287	1.7734	2.1114	2.6087
		0.9	1.5661	1.8738	2.3319	3.0866
	0.2	0.6	1.4531	1.6236	1.8396	2.1218
		0.7	1.5024	1.7170	2.0031	2.4037
		0.8	1.5466	1.8166	2.2010	2.7915
		0.9	1.5756	1.9142	2.4382	3.3572
	0.3	0.6	1.4745	1.6627	1.9060	2.2327
		0.7	1.5223	1.7591	2.0832	2.5537
		0.8	1.5618	1.8597	2.2980	3.0065
		0.9	1.5781	1.9493	2.5488	3.6809
0.4	0.1	0.6	1.3953	1.5262	1.6841	1.8785
		0.7	1.4387	1.5984	1.7980	2.0545
		0.8	1.4815	1.6760	1.9293	2.2727
		0.9	1.5223	1.7591	2.0832	2.5537
	0.2	0.6	1.4242	1.5737	1.7583	1.9919
		0.7	1.4674	1.6495	1.8833	2.1943
		0.8	1.5091	1.7309	2.0291	2.4513
		0.9	1.5466	1.8166	2.2010	2.7915
	0.3	0.6	1.4531	1.6236	1.8396	2.1218
		0.7	1.4955	1.7032	1.9778	2.3581
		0.8	1.5349	1.7878	2.1405	2.6665
		0.9	1.5661	1.8738	2.3319	3.0866

### 3.2 *Comparing*${\hat{\pi }}_{kuk}$*and*${\hat{\pi }}_{p}$

Building on same argument given by inequality (30), the expression to quantify the efficiencies of both estimators is written as,

$$\frac{{\bar{Z}}^{2}\left(\frac{1-f}{n}\right)C_{z}^{2}}{{\left(\mu_{x}-\mu_{y}\right)}^{2}}-\frac{{\bar{Z}}^{2}\left(\frac{1-f}{n}\right)\left(C_{w}^{2}+C_{z}^{2}+2\rho_{wz}C_{w}C_{z}\right)}{{\left(\mu_{x}-\mu_{y}\right)}^{2}}\geq 0\text{,}$$

which, on further simplification leads towards the condition under which ${\hat{\pi }}_{p}$ remain an efficient estimator than ${\hat{\pi }}_{kuk}$.

$$\rho_{zw}\leq -\frac{1}{2}\left(\frac{C_{w}}{C_{z}}\right)\text{,}$$

which, then indicates, that, if $C_{w}\approx C_{z}$, the reduced condition of absolute efficiency of ${\hat{\pi }}_{p}$ over ${\hat{\pi }}_{kuk}$, is $\rho_{zw}\leq -\frac{1}{2}\text{.}$ This is again perfectly aligned with theory of product estimator. Table 2, gathers the numerical evidences to verify the mathematical facts that we established above. Identical performance behavior is witnessed for product estimator as was of ratio estimator with respect to Ref. [13] estimators. The only change in numerical evaluation is the optimal range of correlation coefficient, which now projects moderate negative to strong negative linear relationship between randomized response and auxiliary variable. Thus, based on our study, it is evident that ratio and product estimator are equally efficient but for opposite direction of linear relationship.

**Table 2:** Performance analysis of ${\hat{\pi }}_{P}$ with respect to ${\hat{\pi }}_{kuk}$, in terms of *RE*.

ππ			$\begin{array}{l} \rho_{zw}\\ \theta_{1}\\ \theta_{2} \end{array}$$\rho_{zw}$			
	$\theta_{1}$	$\theta_{2}$	−0.6	−0.7	−0.8	−0.9
0.1	0.1	0.6	1.5024	1.7170	2.0031	2.4037
		0.7	1.5571	1.8454	2.2648	2.9308
		0.8	1.5768	1.9589	2.5857	3.8022
		0.9	1.4755	1.9337	2.8049	5.1044
	0.2	0.6	1.5091	1.7309	2.0291	2.4513
		0.7	1.5618	1.8597	2.2980	3.0065
		0.8	1.5741	1.9672	2.6221	3.9305
		0.9	1.4482	1.9075	2.7936	5.2167
	0.3	0.6	1.5158	1.7450	2.0558	2.5013
		0.7	1.5661	1.8738	2.3319	3.0866
		0.8	1.5700	1.9739	2.6576	4.0660
		0.9	1.4160	1.8734	2.7673	5.2926
0.2	0.1	0.6	1.4674	1.6495	1.8833	2.1943
		0.7	1.5223	1.7591	2.0832	2.5537
		0.8	1.5661	1.8738	2.3319	3.0866
		0.9	1.5741	1.9672	2.6221	3.9305
	0.2	0.6	1.4815	1.6760	1.9293	2.2727
		0.7	1.5349	1.7878	2.1405	2.6665
		0.8	1.5731	1.9012	2.4021	3.2616
		0.9	1.5640	1.9784	2.6915	4.2082
	0.3	0.6	1.4955	1.7032	1.9778	2.3581
		0.7	1.5466	1.8166	2.2010	2.7915
		0.8	1.5774	1.9267	2.4748	3.4587
		0.9	1.5460	1.9796	2.7514	4.5095
0.3	0.1	0.6	1.4314	1.5860	1.7779	2.0227
		0.7	1.4815	1.6760	1.9293	2.2727
		0.8	1.5287	1.7734	2.1114	2.6087
		0.9	1.5661	1.8738	2.3319	3.0866
	0.2	0.6	1.4531	1.6236	1.8396	2.1218
		0.7	1.5024	1.7170	2.0031	2.4037
		0.8	1.5466	1.8166	2.2010	2.7915
		0.9	1.5756	1.9142	2.4382	3.3572
	0.3	0.6	1.4745	1.6627	1.9060	2.2327
		0.7	1.5223	1.7591	2.0832	2.5537
		0.8	1.5618	1.8597	2.2980	3.0065
		0.9	1.5781	1.9493	2.5488	3.6809
0.4	0.1	0.6	1.3953	1.5262	1.6841	1.8785
		0.7	1.4387	1.5984	1.7980	2.0545
		0.8	1.4815	1.6760	1.9293	2.2727
		0.9	1.5223	1.7591	2.0832	2.5537
	0.2	0.6	1.4242	1.5737	1.7583	1.9919
		0.7	1.4674	1.6495	1.8833	2.1943
		0.8	1.5091	1.7309	2.0291	2.4513
		0.9	1.5466	1.8166	2.2010	2.7915
	0.3	0.6	1.4531	1.6236	1.8396	2.1218
		0.7	1.4955	1.7032	1.9778	2.3581
		0.8	1.5349	1.7878	2.1405	2.6665
		0.9	1.5661	1.8738	2.3319	3.0866

### 3.3 *Comparing*${\hat{\pi }}_{kuk}$*and*${\hat{\pi }}_{\text{Re}g}$

Taking on consistent argument built in inequality (35), the efficiency condition is,

$$\frac{\left(\frac{1-f}{n}\right){\bar{Z}}^{2}C_{z}^{2}}{{\left(\mu_{x}-\mu_{y}\right)}^{2}}-\frac{\left(\frac{1-f}{n}\right){\bar{Z}}^{2}C_{z}^{2}\left(1-{\rho_{wz}}^{2}\right)}{{\left(\mu_{x}-\mu_{y}\right)}^{2}}\geq 0\text{,}$$

which, further simplifies to,

(31) $$-1\leq \rho_{zw}\leq 1\text{.}$$

The above inequality (31) directs the fact that regression estimator always remains efficient over the permissible range of correlation co-efficient while comparing with usual [13] estimator. The numerical evaluation of performance of both estimators for various combinations of parameters involved in study is offered by Table 3 and Table 4.

**Table 3:** Performance analysis of ${\hat{\pi }}_{Reg}$ and ${\hat{\pi }}_{Grr}$ with respect to ${\hat{\pi }}_{kuk}$, in terms of *RE*.

π			$\rho_{zw}$											
	$\theta_{1}$	$\theta_{2}$	0.2		0.4		0.6		0.7		0.8		0.9	
			*EFF (Reg)*	*EFF (Grr)*	*EFF (Reg)*	*EFF (Grr)*	*EFF (Reg)*	EFF (Grr)	EFF (Reg)	EFF (Grr)	EFF (Reg)	EFF (Grr)	EFF (Reg)	*EFF (Grr)*
0.1	0.1	0.6	1.0521	1.0603	1.2025	1.2106	1.5782	1.5864	1.9805	1.9887	2.8058	2.8140	5.3163	5.3245
		0.7	1.0521	1.0578	1.2025	1.2081	1.5782	1.5839	1.9805	1.9862	2.8058	2.8114	5.3163	5.3219
		0.8	1.0521	1.0558	1.2025	1.2062	1.5782	1.5819	1.9805	1.9842	2.8058	2.8095	5.3163	5.3200
		0.9	1.0521	1.0543	1.2025	1.2046	1.5782	1.5804	1.9805	1.9827	2.8058	2.8080	5.3163	5.3185
	0.2	0.6	1.0521	1.0600	1.2025	1.2103	1.5782	1.5861	1.9805	1.9884	2.8058	2.8137	5.3163	5.3241
		0.7	1.0521	1.0575	1.2025	1.2078	1.5782	1.5836	1.9805	1.9859	2.8058	2.8112	5.3163	5.3217
		0.8	1.0521	1.0557	1.2025	1.2060	1.5782	1.5818	1.9805	1.9841	2.8058	2.8093	5.3163	5.3198
		0.9	1.0521	1.0542	1.2025	1.2045	1.5782	1.5803	1.9805	1.9826	2.8058	2.8078	5.3163	5.3183
	0.3	0.6	1.0521	1.0597	1.2025	1.2100	1.5782	1.5858	1.9805	1.9881	2.8058	2.8133	5.3163	5.3238
		0.7	1.0521	1.0573	1.2025	1.2076	1.5782	1.5834	1.9805	1.9857	2.8058	2.8109	5.3163	5.3214
		0.8	1.0521	1.0555	1.2025	1.2058	1.5782	1.5816	1.9805	1.9839	2.8058	2.8091	5.3163	5.3196
		0.9	1.0521	1.0540	1.2025	1.2044	1.5782	1.5801	1.9805	1.9824	2.8058	2.8077	5.3163	5.3182
0.2	0.1	0.6	1.0521	1.0622	1.2025	1.2125	1.5782	1.5882	1.9805	1.9906	2.8058	2.8158	5.3163	5.3263
		0.7	1.0521	1.0594	1.2025	1.2097	1.5782	1.5855	1.9805	1.9878	2.8058	2.8130	5.3163	5.3235
		0.8	1.0521	1.0573	1.2025	1.2076	1.5782	1.5834	1.9805	1.9857	2.8058	2.8109	5.3163	5.3214
		0.9	1.0521	1.0557	1.2025	1.2060	1.5782	1.5818	1.9805	1.9841	2.8058	2.8093	5.3163	5.3198
	0.2	0.6	1.0521	1.0614	1.2025	1.2117	1.5782	1.5875	1.9805	1.9898	2.8058	2.8150	5.3163	5.3255
		0.7	1.0521	1.0588	1.2025	1.2091	1.5782	1.5849	1.9805	1.9872	2.8058	2.8125	5.3163	5.3229
		0.8	1.0521	1.0569	1.2025	1.2072	1.5782	1.5829	1.9805	1.9853	2.8058	2.8105	5.3163	5.3210
		0.9	1.0521	1.0553	1.2025	1.2056	1.5782	1.5814	1.9805	1.9837	2.8058	2.8089	5.3163	5.3194
	0.3	0.6	1.0521	1.0607	1.2025	1.2110	1.5782	1.5868	1.9805	1.9891	2.8058	2.8143	5.3163	5.3248
		0.7	1.0521	1.0583	1.2025	1.2086	1.5782	1.5844	1.9805	1.9867	2.8058	2.8119	5.3163	5.3224
		0.8	1.0521	1.0564	1.2025	1.2067	1.5782	1.5825	1.9805	1.9848	2.8058	2.8101	5.3163	5.3206
		0.9	1.0521	1.0550	1.2025	1.2067	1.5782	1.5811	1.9805	1.9834	2.8058	2.8086	5.3163	5.3191
0.3	0.1	0.6	1.0521	1.0644	1.2025	1.2147	1.5782	1.5905	1.9805	1.9928	2.8058	2.8180	5.3163	5.3285
		0.7	1.0521	1.0614	1.2025	1.2117	1.5782	1.5875	1.9805	1.9898	2.8058	2.8150	5.3163	5.3255
		0.8	1.0521	1.0591	1.2025	1.2094	1.5782	1.5852	1.9805	1.9875	2.8058	2.8127	5.3163	5.3232
		0.9	1.0521	1.0573	1.2025	1.2076	1.5782	1.5834	1.9805	1.9857	2.8058	2.8109	5.3163	5.3214
	0.2	0.6	1.0521	1.0630	1.2025	1.2133	1.5782	1.5891	1.9805	1.9914	2.8058	2.8166	5.3163	5.3271
		0.7	1.0521	1.0603	1.2025	1.2106	1.5782	1.5864	1.9805	1.9887	2.8058	2.8140	5.3163	5.3245
		0.8	1.0521	1.0583	1.2025	1.2086	1.5782	1.5844	1.9805	1.9867	2.8058	2.8119	5.3163	5.3224
		0.9	1.0521	1.0566	1.2025	1.2070	1.5782	1.5827	1.9805	1.9850	2.8058	2.8103	5.3163	5.3208
	0.3	0.6	1.0521	1.0618	1.2025	1.2121	1.5782	1.5879	1.9805	1.9902	2.8058	2.8154	5.3163	5.3259
		0.7	1.0521	1.0594	1.2025	1.2097	1.5782	1.5855	1.9805	1.9878	2.8058	2.8130	5.3163	5.3235
		0.8	1.0521	1.0575	1.2025	1.2078	1.5782	1.5836	1.9805	1.9859	2.8058	2.8112	5.3163	5.3217
		0.9	1.0521	1.0560	1.2025	1.2063	1.5782	1.5821	1.9805	1.9859	2.8058	2.8097	5.3163	5.3202
0.4	0.1	0.6	1.0521	1.0672	1.2025	1.2175	1.5782	1.5933	1.9805	1.9956	2.8058	2.8208	5.3163	5.3313
		0.7	1.0521	1.0639	1.2025	1.2142	1.5782	1.5900	1.9805	1.9923	2.8058	2.8175	5.3163	5.3280
		0.8	1.0521	1.0614	1.2025	1.2117	1.5782	1.5875	1.9805	1.9898	2.8058	2.8150	5.3163	5.3255
		0.9	1.0521	1.0594	1.2025	1.2097	1.5782	1.5855	1.9805	1.9878	2.8058	2.8130	5.3163	5.3235
	0.2	0.6	1.0521	1.0649	1.2025	1.2152	1.5782	1.5910	1.9805	1.9933	2.8058	2.8185	5.3163	5.3290
		0.7	1.0521	1.0622	1.2025	1.2125	1.5782	1.5882	1.9805	1.9906	2.8058	2.8158	5.3163	5.3263
		0.8	1.0521	1.0600	1.2025	1.2103	1.5782	1.5861	1.9805	1.9884	2.8058	2.8137	5.3163	5.3241
		0.9	1.0521	1.0583	1.2025	1.2086	1.5782	1.5844	1.9805	1.9867	2.8058	2.8119	5.3163	5.3224
	0.3	0.6	1.0521	1.0630	1.2025	1.2133	1.5782	1.5891	1.9805	1.9914	2.8058	2.8166	5.3163	5.3271
		0.7	1.0521	1.0607	1.2025	1.2110	1.5782	1.5868	1.9805	1.9891	2.8058	2.8143	5.3163	5.3248
		0.8	1.0521	1.0588	1.2025	1.2091	1.5782	1.5849	1.9805	1.9872	2.8058	2.8125	5.3163	5.3229
		0.9	1.0521	1.0573	1.2025	1.2076	1.5782	1.5834	1.9805	1.9857	2.8058	2.8109	5.3163	5.3214

**Table 4:** Performance analysis of ${\hat{\pi }}_{Reg}$ and ${\hat{\pi }}_{Grr}$ with respect to ${\hat{\pi }}_{kuk}$.

π			$\rho_{zw}$											
	$\theta_{1}$	$\theta_{2}$	−0.2		−0.4		−0.6		−0.7		−0.8		−0.9	
			EFF (Reg)	EFF (Grr)	EFF (Reg)	EFF (Grr)	EFF (Reg)	EFF (Grr)	EFF (Reg)	EFF (Grr)	EFF (Reg)	EFF (Grr)	EFF (Reg)	EFF (Grr)
0.1	0.1	0.6	1.0521	1.0603	1.2025	1.2106	1.5782	1.5864	1.9805	1.9887	2.8058	2.8140	5.3163	5.3245
		0.7	1.0521	1.0578	1.2025	1.2081	1.5782	1.5839	1.9805	1.9862	2.8058	2.8114	5.3163	5.3219
		0.8	1.0521	1.0558	1.2025	1.2062	1.5782	1.5819	1.9805	1.9842	2.8058	2.8095	5.3163	5.3200
		0.9	1.0521	1.0543	1.2025	1.2046	1.5782	1.5804	1.9805	1.9827	2.8058	2.8080	5.3163	5.3185
	0.2	0.6	1.0521	1.0600	1.2025	1.2103	1.5782	1.5861	1.9805	1.9884	2.8058	2.8137	5.3163	5.3241
		0.7	1.0521	1.0575	1.2025	1.2078	1.5782	1.5836	1.9805	1.9859	2.8058	2.8112	5.3163	5.3217
		0.8	1.0521	1.0557	1.2025	1.2060	1.5782	1.5818	1.9805	1.9841	2.8058	2.8093	5.3163	5.3198
		0.9	1.0521	1.0542	1.2025	1.2045	1.5782	1.5803	1.9805	1.9826	2.8058	2.8078	5.3163	5.3183
	0.3	0.6	1.0521	1.0597	1.2025	1.2100	1.5782	1.5858	1.9805	1.9881	2.8058	2.8133	5.3163	5.3238
		0.7	1.0521	1.0573	1.2025	1.2076	1.5782	1.5834	1.9805	1.9857	2.8058	2.8109	5.3163	5.3214
		0.8	1.0521	1.0555	1.2025	1.2058	1.5782	1.5816	1.9805	1.9839	2.8058	2.8091	5.3163	5.3196
		0.9	1.0521	1.0540	1.2025	1.2044	1.5782	1.5801	1.9805	1.9824	2.8058	2.8077	5.3163	5.3182
0.2	0.1	0.6	1.0521	1.0622	1.2025	1.2125	1.5782	1.5882	1.9805	1.9906	2.8058	2.8158	5.3163	5.3263
		0.7	1.0521	1.0594	1.2025	1.2097	1.5782	1.5855	1.9805	1.9878	2.8058	2.8130	5.3163	5.3235
		0.8	1.0521	1.0573	1.2025	1.2076	1.5782	1.5834	1.9805	1.9857	2.8058	2.8109	5.3163	5.3214
		0.9	1.0521	1.0557	1.2025	1.2060	1.5782	1.5818	1.9805	1.9841	2.8058	2.8093	5.3163	5.3198
	0.2	0.6	1.0521	1.0614	1.2025	1.2117	1.5782	1.5875	1.9805	1.9898	2.8058	2.8150	5.3163	5.3255
		0.7	1.0521	1.0588	1.2025	1.2091	1.5782	1.5849	1.9805	1.9872	2.8058	2.8125	5.3163	5.3229
		0.8	1.0521	1.0569	1.2025	1.2072	1.5782	1.5829	1.9805	1.9853	2.8058	2.8105	5.3163	5.3210
		0.9	1.0521	1.0553	1.2025	1.2056	1.5782	1.5814	1.9805	1.9837	2.8058	2.8089	5.3163	5.3194
	0.3	0.6	1.0521	1.0607	1.2025	1.2110	1.5782	1.5868	1.9805	1.9891	2.8058	2.8143	5.3163	5.3248
		0.7	1.0521	1.0583	1.2025	1.2086	1.5782	1.5844	1.9805	1.9867	2.8058	2.8119	5.3163	5.3224
		0.8	1.0521	1.0564	1.2025	1.2067	1.5782	1.5825	1.9805	1.9848	2.8058	2.8101	5.3163	5.3206
		0.9	1.0521	1.0550	1.2025	1.2067	1.5782	1.5811	1.9805	1.9834	2.8058	2.8086	5.3163	5.3191
0.3	0.1	0.6	1.0521	1.0644	1.2025	1.2147	1.5782	1.5905	1.9805	1.9928	2.8058	2.8180	5.3163	5.3285
		0.7	1.0521	1.0614	1.2025	1.2117	1.5782	1.5875	1.9805	1.9898	2.8058	2.8150	5.3163	5.3255
		0.8	1.0521	1.0591	1.2025	1.2094	1.5782	1.5852	1.9805	1.9875	2.8058	2.8127	5.3163	5.3232
		0.9	1.0521	1.0573	1.2025	1.2076	1.5782	1.5834	1.9805	1.9857	2.8058	2.8109	5.3163	5.3214
	0.2	0.6	1.0521	1.0630	1.2025	1.2133	1.5782	1.5891	1.9805	1.9914	2.8058	2.8166	5.3163	5.3271
		0.7	1.0521	1.0603	1.2025	1.2106	1.5782	1.5864	1.9805	1.9887	2.8058	2.8140	5.3163	5.3245
		0.8	1.0521	1.0583	1.2025	1.2086	1.5782	1.5844	1.9805	1.9867	2.8058	2.8119	5.3163	5.3224
		0.9	1.0521	1.0566	1.2025	1.2070	1.5782	1.5827	1.9805	1.9850	2.8058	2.8103	5.3163	5.3208
	0.3	0.6	1.0521	1.0618	1.2025	1.2121	1.5782	1.5879	1.9805	1.9902	2.8058	2.8154	5.3163	5.3259
		0.7	1.0521	1.0594	1.2025	1.2097	1.5782	1.5855	1.9805	1.9878	2.8058	2.8130	5.3163	5.3235
		0.8	1.0521	1.0575	1.2025	1.2078	1.5782	1.5836	1.9805	1.9859	2.8058	2.8112	5.3163	5.3217
		0.9	1.0521	1.0560	1.2025	1.2063	1.5782	1.5821	1.9805	1.9859	2.8058	2.8097	5.3163	5.3202
0.4	0.1	0.6	1.0521	1.0672	1.2025	1.2175	1.5782	1.5933	1.9805	1.9956	2.8058	2.8208	5.3163	5.3313
		0.7	1.0521	1.0639	1.2025	1.2142	1.5782	1.5900	1.9805	1.9923	2.8058	2.8175	5.3163	5.3280
		0.8	1.0521	1.0614	1.2025	1.2117	1.5782	1.5875	1.9805	1.9898	2.8058	2.8150	5.3163	5.3255
		0.9	1.0521	1.0594	1.2025	1.2097	1.5782	1.5855	1.9805	1.9878	2.8058	2.8130	5.3163	5.3235
	0.2	0.6	1.0521	1.0649	1.2025	1.2152	1.5782	1.5910	1.9805	1.9933	2.8058	2.8185	5.3163	5.3290
		0.7	1.0521	1.0622	1.2025	1.2125	1.5782	1.5882	1.9805	1.9906	2.8058	2.8158	5.3163	5.3263
		0.8	1.0521	1.0600	1.2025	1.2103	1.5782	1.5861	1.9805	1.9884	2.8058	2.8137	5.3163	5.3241
		0.9	1.0521	1.0583	1.2025	1.2086	1.5782	1.5844	1.9805	1.9867	2.8058	2.8119	5.3163	5.3224
	0.3	0.6	1.0521	1.0630	1.2025	1.2133	1.5782	1.5891	1.9805	1.9914	2.8058	2.8166	5.3163	5.3271
		0.7	1.0521	1.0607	1.2025	1.2110	1.5782	1.5868	1.9805	1.9891	2.8058	2.8143	5.3163	5.3248
		0.8	1.0521	1.0588	1.2025	1.2091	1.5782	1.5849	1.9805	1.9872	2.8058	2.8125	5.3163	5.3229
		0.9	1.0521	1.0573	1.2025	1.2076	1.5782	1.5834	1.9805	1.9857	2.8058	2.8109	5.3163	5.3214

### 3.4 *Comparing*${\hat{\pi }}_{kuk}$*and*${\hat{\pi }}_{Grr}$

On comparing equations (4), (29), we obtained the efficiency condition as,

$$\frac{{\bar{Z}}^{2}\lambda C_{z}^{2}}{{\left(\mu_{x}-\mu_{y}\right)}^{2}}-\frac{{\bar{Z}}^{2}C_{z}^{2}\left(1-\rho_{zw}^{2}\right)\lambda \left(1-\lambda C_{w}^{2}\right)}{{\left(\mu_{X}-\mu_{Y}\right)}^{2}\left(C_{z}^{2}\left(1-\rho_{zw}^{2}\right)\lambda +\left(1-\lambda C_{w}^{2}\right)\right)}\geq 0\text{.}$$

It is verifiable that, above inequality further simplifies to,

$$\left(C_{z}^{2}\left(1-\rho_{zw}^{2}\right)\lambda +\left(1-\lambda C_{w}^{2}\right)\right)-\left(1-\rho_{zw}^{2}\right)\left(1-\lambda C_{w}^{2}\right)\geq 0\text{,}$$

which is then written as,

(32) $$-\frac{\sqrt{\lambda }C_{z}}{\sqrt{\lambda \left(C_{z}^{2}+C_{w}^{2}\right)-1}}\leq \rho_{zw}\leq \frac{\sqrt{\lambda }C_{z}}{\sqrt{\lambda \left(C_{z}^{2}+C_{w}^{2}\right)-1}}\text{.}$$

The inequality (32) provides the efficiency condition in the form of optimal range of correlation coefficient over which the generalized regression-cum-ratio estimator performs better than usual [13] estimator. The numerical findings of comparative efficiency analysis are compiled in Table 3, Table 4, for both, the regression and the generalized regression-cum-ratio estimators, while considering [13] as benchmark.

The finding presented in above Table 3, Table 4 highlighted the fact that, the regression and generalized regression-cum-ratio estimators are capable of accommodating the desired characteristics of both ratio and product estimators with extended utility. The estimators remain efficient with respect to Ref. [13] estimator even when linear relationship between auxiliary variable and randomized response is weak. The generalized regression-cum-ratio estimator performs comparatively better, than the regression estimator over the permissible range of correlation coefficient and for all considered combinations of parameters. Moreover, as the strength of linear relationship increases (positive direction or negative direction), the proposed estimator's performance becomes more vivid.

## 4 Employing the shrinkage estimation procedures to incorporate the crude Prior Estimate of π

In many situations of practical importance, investigators may have the prior knowledge regarding the parameter, π, population proportion of sensitive characteristics, either through the historical track of alike studies or based on expert opinion. Moving further from incorporating the auxiliary information, now we explore the applicability of approximate value (or crude prior estimate) of, π. That is, we plan to apply shrinkage estimation. As mentioned by Ref. [51], shrinkage estimation techniques have been applied widely in many disciplines. Shrinkage estimation is mainly based on the premise of using a weighted average between one high-dimensional estimator such as maximum likelihood estimator or moment estimator with smaller accuracy and precision, and one low-dimensional estimator having larger accuracy and precision. The major issues concerning shrinkage estimation are finding a low dimensional estimator that is appropriate for a given estimation study, and to decide upon the shrinkage weight. In the literature many shrinkage estimation techniques have been proposed by different studies including [52,53,46,47]. The current study focuses on two pioneer shrinkage estimation procedures by Refs. [46,20]. In this section, we derive the mathematical expressions for bias, MSE of estimators and optimal ranges for weights inducing the extent of prior information, when both methods are applied to enhance estimation of sensitive proportion in survey research.

### 4.1 Searls procedure

The general form of new hybrid estimator when following [46] method is written as,

$${\hat{\pi }}_{S}=k_{3}{\hat{\pi }}_{kuk}\text{,}$$

where, $k_{3}$ is weight, taking positive values less than or equal to 1.

#### 4.1.1 Bias and MSE of the estimator

The bias of estimator, ${\hat{\pi }}_{S}$, is given as,

$$E\left({\hat{\pi }}_{S}\right)-\pi =\pi \left(k_{3}-1\right)\text{.}$$

The above equation establishes that the [46] procedure leads towards a biased estimator depending on the value of induced weight. Further, MSE of the estimator is evaluated as under,

(35) $$MSE\left({\hat{\pi }}_{S}\right)=k_{3}^{2}Var\left({\hat{\pi }}_{kuk}\right)+\pi^{2}\left(k_{3}-1\right)\text{,}$$

where, variance of [13] estimator is given in equation (4).

#### 4.1.2 *Quantifying the Optimal range of*$k_{3}$

Now, we evaluate the range of weight, $k_{3}$, for which Searls’ procedure performs more efficiently than usual [13] estimator. The optimal range is evaluated by following the argument of inequality (30), which on simplification provides us,

(36) $$k_{3}^{2}\left(-Var\left({\hat{\pi }}_{kuk}\right)-\pi^{2}\right)+k_{3}\left(2\pi^{2}\right)+\left(Var\left({\hat{\pi }}_{kuk}\right)-\pi^{2}\right)\geq 0\text{.}$$

On solving the above quadratic equation, we obtain the lower and upper limits of optimal range of $k_{3}$, as follows,

(37) $$\frac{\pi^{2}-Var\left({\hat{\pi }}_{kuk}\right)}{\pi^{2}+Var\left({\hat{\pi }}_{kuk}\right)}\leq k_{3}\leq 1\text{.}$$

The expression derived in inequality (37), reduces to the lower and upper limits values for given combinations of parameters, where the [46] method of aiding data based estimator performs better than [13] estimator. Table 5, comprehends the numerical results evaluated using inequality (37) for various combinations of parameters involved in study.

**Table 5:** Optimal ranges for weights while employing [46,47] approaches.

π			Searls		Thompson		π			Searls		Thompson	
	$\theta_{1}$	$\theta_{2}$	$k_{3L}$	$k_{3U}$	$k_{4L}$	$k_{4U}$		$\theta_{1}$	$\theta_{2}$	$k_{3L}$	$k_{3U}$	$k_{4L}$	$k_{4U}$
0.1	0.1	0.6	0.00502	1	0	1.0000	0.2	0.1	0.6	0.60000	1	0	1.0000
		0.7	0.21951	1	0	1.0000			0.7	0.71060	1	0	1.0000
		0.8	0.42628	1	0	1.0000			0.8	0.79454	1	0	1.0000
		0.9	0.62519	1	0	0.95968			0.9	0.86019	1	0	1.0000
	0.2	0.6	0.0000	1	0	1.0000		0.2	0.6	0.43884	1	0	1.0000
		0.7	0.04712	1	0	1.0000			0.7	0.61290	1	0	1.0000
		0.8	0.30340	1	0	1.0000			0.8	0.73745	1	0	1.0000
		0.9	0.55284	1	0	1.0000			0.9	0.82972	1	0	1.0000
	0.3	0.6	0.45631	1	0	1.0000		0.3	0.6	0.18343	1	0	1.0000
		0.7	0.0000	1	0	1.0000			0.7	0.46185	1	0	1.0000
		0.8	0.14285	1	0	1.0000			0.8	0.65289	1	0	1.0000
		0.9	0.45631	1	0	1.0000			0.9	0.78704	1	0	1.0000
π			Searls		Thompson		π			Searls		Thompson	
	$\theta_{1}$	$\theta_{2}$	$k_{3L}$	$k_{3U}$	$k_{4L}$	$k_{4U}$		$\theta_{1}$	$\theta_{2}$	$k_{3L}$	$k_{3U}$	$k_{4L}$	$k_{4U}$
0.3	0.1	0.6	0.80180	1	0	0.61111	0.4	0.1	0.6	0.88679	1	0	0.26629
		0.7	0.85694	1	0	0.47112			0.7	0.91731	1	0	0.19884
		0.8	0.89599	1	0	0.35986			0.8	0.93829	1	0	0.15072
		0.9	0.92500	1	0	0.26964			0.9	0.95352	1	0	0.11480
	0.2	0.6	0.70454	1	0	0.81889		0.2	0.6	0.82440	1	0	0.39537
		0.7	0.80180	1	0	0.61111			0.7	0.88235	1	0	0.27586
		0.8	0.86442	1	0	0.45065			0.8	0.91795	1	0	0.19740
		0.9	0.90748	1	0	0.32497			0.9	0.94165	1	0	0.14287
	0.3	0.6	0.52844	1	0	1.0000		0.3	0.6	0.70454	1	0	0.61470
		0.7	0.71062	1	0	0.80715			0.7	0.82310	1	0	0.39794
		0.8	0.81634	1	0	0.57594			0.8	0.88679	1	0	0.26629
		0.9	0.88284	1	0	0.39857			0.9	0.18137	1	0	0.92500

### 4.2 Thompson procedure

Another shrinkage estimation technique was introduced by Ref. [47] to aid the data based estimator through crude prior point estimator of parameter of interest, evaluated using prior knowledge through expert opinion or historical track of the study. Let us denote $\pi_{o}$ as crude prior point estimate of population proportion of sensitive attribute, π. For evaluation purposes, the value of prior estimate, $\pi_{o}$, is fixed at 0.15 throughout the current study. The hybrid estimator, ${\hat{\pi }}_{T}$, to estimate the proportion of sensitive attribute based on approximated value $\pi_{o}$ and data oriented ${\hat{\pi }}_{kuk}$, given in equation (3), is written as,

$${\hat{\pi }}_{T}=k_{4}\pi_{o}+\left(1-k_{4}\right){\hat{\pi }}_{kuk}\text{,}$$

where, $k_{4}$ is the weight, indicating the extent to which crude prior estimate, ranging from 0 to 1, is allowed to influence the study in hand. A value of $k_{4}$ closer to 0 is a case when investigator tends to rely on data based estimator, whereas, the deviations from 0 project the higher extent of given role of the prior estimate in study.

#### 4.2.1 Bias and MSE of the estimator

The bias is found by employing the elementary expression and we get,

(38) $$bias\left({\hat{\pi }}_{T}\right)=k_{4}\left(\pi_{0}-\pi \right)\text{.}$$

equation (38), establishes the fact that involving the crude prior point estimate, we obtain a biased estimate of population proportion of sensitive attribute. The mathematical expression of MSE of the estimator is obtained as,

(39) $$MSE\left({\hat{\pi }}_{T}\right)=\text{var}\left({\hat{\pi }}_{T}\right)+bias^{2}\left({\hat{\pi }}_{Cpe}\right)\text{.}$$

By using equations (38), (39), it can be written as,

(40) $$MSE\left({\hat{\pi }}_{T}\right)={\left(1-k_{4}\right)}^{2}\left(Var\left({\hat{\pi }}_{kuk}\right)\right)+k_{4}^{2}{\left(\pi_{o}-\pi \right)}^{2}\text{.}$$

#### 4.2.2 *Quantifying the Optimal range of*$k_{4}$

The optimal range for $k_{4}$ is obtained by comparing equation (4) and equation (40), for efficiency evaluation of Thomson procedure with respect to Ref. [13] estimation technique. On comparison, we have a quadratic equation of following form,

(41) $$k_{4}^{2}\left(-{\left(\pi_{o}-\pi \right)}^{2}-Var\left({\hat{\pi }}_{kuk}\right)\right)+k_{4}\left(2Var\left({\hat{\pi }}_{kuk}\right)\right)\geq 0\text{,}$$

which, on solving gives the lower and upper limits of the ranges of $k_{4}$, where Thomson procedure proves to be more effective than [13] estimator. The optimal range is,

(42) $$0\leq k_{4}\leq \frac{2Var\left({\hat{\pi }}_{kuk}\right)}{{\left(\pi_{o}-\pi \right)}^{2}+Var\left({\hat{\pi }}_{kuk}\right)}\text{.}$$

Table 5, compiles the numerical values of lower and upper limits of optimal ranges based on (37), (42) when [46,47], procedures are in effect, respectively.

Table 5, compiles the numerical results providing lower and upper limits of the optimal ranges of shrinkage weights with respect to various combinations of parameters influencing the study. It is noted, in the case of [47] procedure, the values of upper limit show tendency to fall outside the permissible range of weight, that is, 0 and 1. Interestingly, this behavior is specifically attributed with the cases when the true population parameter, π, and crude point estimator, $\pi_{o}$, lie closer to each other, that is, when π=0.1,0.2. This estimation disguise can in fact be used as indication if the point estimator is closer to true population parameter. This procedural limitation can also be noticed through mathematical expression given in inequality (42); closer the difference between $\pi_{o}$ and π, smaller will be the denominator of equation which then leads to un-interpretable limiting values of optimal weights. To overcome this issue, those limiting values are replaced by 1 thus in relevant cases it is considered that optimal weight value lies over full permissible range.

## 5 Mathematical and numerical evaluation of the performance of estimators whileEmploying shrinkage estimation procedures

In this section, we explore the performance of [13] estimator when it is aided through shrinkage estimation procedures. We derive the efficiency conditions while considering the optimal values and mid-point values of weights ($k_{3}$ and $k_{4}$) over optimal range (see equations (37), (42)), for both Searls and Thompson techniques. The performance analysis is then conducted for both shrinkage techniques with respect to Ref. [13] estimator. Table 6 is being dedicated to comprehend the numerical findings while considering wide range of combinations of values of parameters.

**Table 6:** Relative efficiency of ${\hat{\pi }}_{kuk}$ when aided by optimal values and mid-range values of weights while employing [46,47] approaches.

π			Searls		Thompson	π			Searls		Thompson
	$\theta_{1}$	$\theta_{2}$	$k_{Sopt}$	$k_{Smid}$	$k_{Topt}$		$\theta_{1}$	$\theta_{2}$	$k_{Sopt}$	$k_{Smid}$	$k_{Topt}$
0.1	0.1	0.6	1.9900	1.0099	4.9600	0.2	0.1	0.6	1.2500	0.2711	5.0000
		0.7	1.6400	0.6529	3.5600			0.7	1.1691	0.1835	3.7066
		0.8	1.4022	0.4103	2.6089			0.8	1.1144	0.1242	2.8318
		0.9	1.2306	0.2353	1.9225			0.9	1.0751	0.0815	2.2025
	0.2	0.6	2.5400	1.5710	7.1600		0.2	0.6	1.3900	0.4228	7.2400
		0.7	1.9100	0.9283	4.6400			0.7	1.2400	0.2603	4.8400
		0.8	1.5344	0.5452	3.1377			0.8	1.1511	0.1639	3.4177
		0.9	1.2879	0.2937	2.1518			0.9	1.0930	0.1009	2.4889
	0.3	0.6	3.7233	2.7778	11.8933		0.3	0.6	1.6900	0.7478	12.0400
		0.7	2.4025	1.4307	6.6100			0.7	1.3681	0.3991	6.8900
		0.8	1.7500	0.7651	4.0000			0.8	1.2100	0.2277	4.3600
		0.9	1.3733	0.3808	2.4933			0.9	1.1191	0.1292	2.9066
π			Searls		Thompson	π			Searls		Thompson
	$\theta_{1}$	$\theta_{2}$	$k_{Sopt}$	$k_{Smid}$	$k_{Topt}$		$\theta_{1}$	$\theta_{2}$	$k_{Sopt}$	$k_{Smid}$	$k_{Topt}$
0.3	0.1	0.6	1.1100	0.1326	1.4400	0.4	0.1	0.6	1.0600	0.0848	1.1536
		0.7	1.0770	0.0929	1.3081			0.7	1.0431	0.0610	1.1104
		0.8	1.0548	0.0662	1.2194			0.8	1.0318	0.0450	1.0815
		0.9	1.0389	0.0470	1.1558			0.9	1.0237	0.0336	1.0609
	0.2	0.6	1.1733	0.2089	1.6933		0.2	0.6	1.0962	0.1359	1.2464
		0.7	1.1100	0.1326	1.4400			0.7	1.0625	0.0883	1.1600
		0.8	1.0727	0.0877	1.2908			0.8	1.0427	0.0605	1.1095
		0.9	1.0485	0.0585	1.1940			0.9	1.0300	0.0425	1.0769
	0.3	0.6	1.3085	0.3714	2.2340		0.3	0.6	1.1733	0.2441	1.4437
		0.7	1.1691	0.2039	1.6766			0.7	1.0970	0.1370	1.2484
		0.8	1.1011	0.1219	1.4044			0.8	1.0600	0.0848	1.1536
		0.9	1.0622	0.0750	1.2488			0.9	1.0389	0.0551	1.0997

### 5.1 Quantifying the efficiency conditions for searls procedure

Now, we derive the expressions to evaluate the efficiency conditions for Searls shrinkage estimation procedure while using optimal value and mid-range value of $k_{3}$, over the range offered by equation (37).

#### 5.1.1 *Efficiency Condition and optimal*$k_{3}$

The optimal value of $k_{3}$ is obtained by differentiating equation (35) with respect to $k_{3}$ and then equating it to 0, we get,

(43) $$k_{3\left(opt\right)}=\frac{\pi^{2}}{Var\left({\hat{\pi }}_{kuk}\right)+\pi^{2}}\text{.}$$

The efficiency condition is established by using optimal value of (43) in inequality (36), and we attained,

(44) $$Eff\left({\hat{\pi }}_{Sopt}\right)=\frac{Var\left({\hat{\pi }}_{kuk}\right)+\pi^{2}}{\pi^{2}}$$

equation (44) demonstrates the fact that the Searls’ shrinkage procedure will always lead towards efficient estimation of proportions of sensitive attribute using the optimal value of $k_{3}$.

#### 5.1.2 *Efficiency Condition and mid-range*$k_{3}$

The mid-range value of $k_{3}$ is found from inequality (37); the value is,

(45) $$k_{3mid}=\pi^{2}$$

To achieve the efficiency condition under mid-range value (equation (45)), we use this value in inequality (36) and get,

$$\frac{Var\left({\hat{\pi }}_{kuk}\right)}{\pi^{2}\left(\pi^{2}\left(Var\left({\hat{\pi }}_{kuk}\right)+\pi^{2}-2\right)+1\right)}\geq 1$$

### 5.2 Quantifying the efficiency conditions for thompson procedure

It can be mathematically verified that for Thompson procedure, optimal value and mid-range value of weight $k_{4}$ are equal. Then, the optimal value of $k_{4}$ is attained by differentiating equation (40) and equating it to zero; we get,

$$k_{4\left(opt\right)}=\frac{Var\left({\hat{\pi }}_{kuk}\right)}{Var\left({\hat{\pi }}_{kuk}\right)+{\left(\pi_{o}-\pi \right)}^{2}}\text{.}$$

By employing the above optimal $k_{4}$ value in inequality (41), we obtained the simplified version to evaluate the efficiency of shrinkage based estimator with respect to optimal weight, which is,

(46) $$Eff\left({\hat{\pi }}_{Topt}\right)=\frac{Var\left({\hat{\pi }}_{kuk}\right)+{\left(\pi_{o}-\pi \right)}^{2}}{{\left(\pi_{o}-\pi \right)}^{2}}$$

The (46) enforces that the crude prior based estimate ${\hat{\pi }}_{T}$, under the optimal weight, will always provide efficient estimator compared to usual [13] estimator, as long as $Var\left({\hat{\pi }}_{kuk}\right)\geq 0$ (which it must be). Next, we present Table 6, containing a detailed account highlighting various aspects of numerical evaluations for considered shrinkage estimation strategies.

The numerical results compiled in Table 6 reveal that the [47] procedure provides efficient estimator than [46] shrinkage approach with respect to Ref. [13] method. It is also witnessed that the impact of crude prior estimate in case of Thompson procedure has noticeable effect on performance; as the difference between true value of population parameter and point estimate increases, the performance of the estimator based on [47] method decreases but it still remains more efficient than contemporary approaches while considering the optimal value of weight. Furthermore, at mid-range value of weight [46], based estimator produce less efficient results than usual [13] estimator (mostly); for comparative purposes the relevant values of performance analysis are documented and highlighted in the table above for all combinations of parameters in the study.

## 6 Discussion and future research aspects

In this paper, we further explored the applicability of classic [13] estimator to estimate the proportion of sensitive characteristics in survey research when additional information about the parameter of interest is available. The utility of Kuk's estimator is investigated in these scenarios while aiding the study parameter through auxiliary variable approach and shrinkage estimation procedures. For the study purposes, we considered four commonly used auxiliary information based estimators and two classic shrinkage estimation approaches. This paper establishes the mathematics required to meet the objectives as well as a detailed numerical investigation of comparative performances is also offered for various combinations of parameters to the readers to get a clearer picture of the issue. Based on numerical and mathematical evidences, it is observed that generalized regression-cum-ratio estimator stands out among the class of auxiliary information based techniques, whereas, Thompson shrinkage estimation approach outperforms the Searls procedure. Numerical evaluations of performance analysis delineate that the most efficient results are noted in the case of using optimal weights values when dealing with shrinkage estimation procedures. The role of the extent of randomization induced in data through randomized response technique is well noticed in our case. Higher the extent of randomization in data less efficient are the employed estimators, even though they still remain more efficient than usual Kuk's estimator. In case of Thompson shrinkage procedure, crude prior point estimator becomes another stack holder; as our prior estimate value moves away from the true value of population parameter the performance of proposed estimator declines. It will be interesting to see the behavior of proposed methods in more complicated extensions such as in the case of two phase sampling. Another future aspect is to explore the applicability of considered approaches when auxiliary variable is also abstracted through randomized response techniques. Furthermore, in many situation of practical importance, instead of having the leisure to access the full data, only partial data are available; based on these methods, exploring the links between masked data and randomized response technique and thus developing a common frame for handling these issues, is also worth studying. The proposed estimators may also be explored using successive sampling and ranked set sampling. Utilizing the proposed estimators in complex surveys would attract survey statisticians.

## Data availability

Data included in article/supplementary material/referenced in article.

## CRediT authorship contribution statement

**Zawar Hussain:** Writing – review & editing, Writing – original draft, Visualization, Validation, Software, Resources, Methodology, Investigation, Formal analysis, Data curation, Conceptualization. **Ishtiaq Hussain:** Writing – review & editing, Writing – original draft, Visualization, Validation, Software, Methodology, Investigation, Formal analysis, Data curation, Conceptualization. **Salman A. Cheema:** Writing – review & editing, Writing – original draft, Visualization, Validation, Software, Resources, Methodology, Formal analysis, Data curation, Conceptualization. **Kalim Ullah:** Writing – review & editing, Writing – original draft, Visualization, Validation, Software, Methodology, Investigation, Formal analysis, Data curation, Conceptualization. **Sultan Salem:** Writing – review & editing, Writing – original draft, Visualization, Validation, Software, Methodology, Investigation, Formal analysis, Data curation, Conceptualization. **Walid Emam:** Writing – review & editing, Writing – original draft, Visualization, Validation, Software, Methodology, Investigation, Formal analysis, Data curation, Conceptualization. **Yusra Tashkandy:** Conceptualization, Data curation, Formal analysis, Investigation, Methodology, Resources, Software, Validation, Visualization, Writing – original draft, Writing – review & editing.

## Declaration of competing interest

The authors declare that they have no known competing financial interests or personal relationships that could have appeared to influence the work reported in this paper.
